# Spatial Cognition in Children With Physical Disability; What Is the Impact of Restricted Independent Exploration?

**DOI:** 10.3389/fnhum.2021.669034

**Published:** 2021-09-16

**Authors:** Emily K. Farran, Valerie Critten, Yannick Courbois, Emma Campbell, David Messer

**Affiliations:** ^1^School of Psychology, University of Surrey, Guildford, United Kingdom; ^2^Faculty of Wellbeing, Education and Language Studies, The Open University, Milton Keynes, United Kingdom; ^3^ULR 4072 – Psychologie: Interactions Temps Émotions Cognition, Université de Lille, Lille, France; ^4^UCL Institute of Education, London, United Kingdom

**Keywords:** physical disability, learning difficulties, spatial cognition, motor, navigation, cerebral palsy

## Abstract

Given the developmental inter-relationship between motor ability and spatial skills, we investigated the impact of physical disability (PD) on spatial cognition. Fifty-three children with special educational needs including PD were divided into those who were wheelchair users (*n* = 34) and those with independent locomotion ability (*n* = 19). This division additionally enabled us to determine the impact of limited independent physical exploration (i.e., required wheelchair use) on spatial competence. We compared the spatial performance of children in these two PD groups to that of typically developing (TD) children who spanned the range of non-verbal ability of the PD groups. Participants completed three spatial tasks; a mental rotation task, a spatial programming task and a desktop virtual reality (VR) navigation task. Levels of impairment of the PD groups were broadly commensurate with their overall level of non-verbal ability. The exception to this was the performance of the PD wheelchair group on the mental rotation task, which was below that expected for their level of non-verbal ability. Group differences in approach to the spatial programming task were evident in that both PD groups showed a different error pattern from the TD group. These findings suggested that for children with both learning difficulties and PD, the unique developmental impact on spatial ability of having physical disabilities, over and above the impact of any learning difficulties, is minimal.

## Introduction

Spatial cognition involves perceiving the location, dimension and properties of objects and their relationships to one another; it is core to everyday living, e.g., reading maps, packing a suitcase. There is a known relationship between motor competence and spatial cognition. For example, in typical infants, the emergence of independent walking predicts the development of spatial understanding about the layout of their environment (Clearfield, 2004) and locomotor experience in infancy enhances spatial cognition (Yan et al., 1998). This is supported by longitudinal evidence that the age at which walking emerges is predictive of spatial cognition at 32 months (Oudgenoeg-Paz et al., 2015). Beyond infancy, an association has been shown between motor ability and mental rotation performance in 5- to 6-year-olds (Jansen and Heil, 2010), and between motor ability and spatial navigation performance in 5- to 11-year-olds (Farran et al., 2019).

Further evidence for the relationship between the motor and spatial domains comes from individuals with physical disability (PD), including those with Cerebral Palsy. Physical Disability is a disturbance of movement and is used as an umbrella term that includes various subtypes and causal pathways. A diagnosis of Cerebral Palsy is given when the disorder of movement results from an early acquired non-progressive brain lesion (Rosenbaum et al., 2006); individuals with Cerebral Palsy also present with varied neural presentation and cognitive impairments (Ego et al., 2015; Stadskleiv et al., 2017). Stadskleiv et al. (2017) report that the majority of individuals with Cerebral Palsy in their study presented with white matter lesions. Their measure of MRI presentation was not associated with motor outcome, but was associated with level of cognitive ability.

Studies that have specifically investigated spatial cognition in children with PD have shown that this group demonstrate impaired spatial knowledge of their environment (Stanton et al., 2002; Wiedenbauer and Jansen-Osmann, 2006), and that individuals with PD present with impaired visuo-spatial perception (Stiers et al., 2002; Critten et al., 2018). In children with Cerebral Palsy, Belmonti et al. (2015) report impaired spatial memory on a table-top task and a large-scale spatial memory task. They also report an association between spatial memory and the extent of neural impairment for right-hemisphere lesions, but not for left-hemisphere lesions. They explain this with respect to evidence for right lateralization of visuospatial functions (for example, the right inferior parietal lobe; Schintu et al., 2014) and perception of self-motion (right parietal–temporal areas; Dieterich et al., 2003).

The aim of the current study was to better understand the relationship between motor and spatial ability domains by further investigating the impact of physical disability on spatial cognition while also contributing to the limited literature describing the impact of independent physical exploration on children’s sense of spatial competence. With reference to physical exploration, Oudgenoeg-Paz et al. (2015) reported that scores on a self-locomotion physical exploration measure among their typically developing young participants (20 months of age) was predictive of small scale spatial cognition at 32 months [assessed with the Block Design subtest of the Wechsler Intelligence Scales for Children – Fourth Edition (WISC-IV); Wechsler, 2003]. Furthermore, investigators have shown that a child’s experience of physical exploration in their local environment is related to the development of strategies required for successful navigation of space (Cornell et al., 2001). Since physical exploration is likely to be restricted in those with PD, due to their poor motor co-ordination, muscular weakness, limited sensations such as paralysis, difficulties with proprioception (perception of the body) and/or poor balance (Sit et al., 2007), comparing spatial cognition skills in children with PD and children without PD, can provide potential insight into the role of physical exploration opportunity as a causal factor in the development of spatial cognition.

In this study we focus on two groups of children with special educational needs including PD: children with PD who are wheelchair users; and those with PD who have independent locomotion. These groups differ with respect to independent exploration because restrictions on exploration are likely to be increased for wheelchair users, especially in the early years. This is because, for wheelchair users, some activities and places are inaccessible and, although there are wheelchair users who are able to self-propel, many wheelchair users are often guided along routes by helpers who may repeat the same routes. This limits the individual’s active control over their exploration. Active control was investigated by Foreman et al. (1994) who demonstrated poorer performance in a radial search task in 6-year-olds who were trained passively compared to 6-year-olds who experienced active training. For both passive and active free-choice conditions, they included a walking and a sitting (being pushed in a push chair) condition. They determined that the free-choice element, i.e., self-initiated exploration, was more important than the type of locomotion, thus emphasizing that for wheelchair uses, restrictions to their autonomy of movement can negatively impact spatial cognition.

Whilst the above review demonstrates an incomplete understanding of the relationship between motor competence and spatial reasoning, there is a consistent pattern of past findings showing an association between them. To our knowledge, our study provides the first investigation of the relationship between motor impairments and small- and large-scale spatial cognition in a large group of children with PD. We included three assessments of spatial cognition. First, we used a mental rotation task, a relatively pure measure of small-scale spatial ability with no physical manipulation requirements in which participants match a rotated image to one of two mirror-imaged upright images. Uttal et al. (2013) and Newcombe (2018) refer to mental rotation as requiring intrinsic spatial coding, i.e., the within-object spatial relations that constitute the structure of the object. This spatial task activates the posterior parietal cortex (Zacks, 2008). It also taps into processes that are common to motor activity (Wohlschläger and Wohlschläger, 1998), and activates the precentral sulcus, a neural area associated with motor activity (Zacks, 2008). Particularly relevant to the current study, this brain activation from a mental rotation task is atypical in individuals with impaired motor ability (e.g., Biotteau et al., 2016; Kashuk et al., 2017). We predicted impaired mental rotation abilities in our participants with PD, relative to those with typical development and suspected that this deficit would be more evident among children with PD who were wheelchair users (for whom exploration might have been relatively limited) than among children with PD who were able to walk independently – as exploration was found to be associated with small scale spatial performance (Oudgenoeg-Paz et al., 2015).

We also included two route learning tasks; in contrast to the mental rotation task, these tasks can be classified as extrinsic spatial tasks (Uttal et al., 2013; Newcombe, 2018), i.e., requiring coding of the spatial relations between objects. The spatial programming task was a 2D route learning problem presented via a freely available Bee-Bot App. Bee-Bots are programmable robots and the Bee-Bot app was presented to children on an iPad. Participants were shown a map-like viewer-independent/allocentric perspective and asked to program the route that the Bee-Bot should take in order to arrive at a flower. This form of presentation allows the participant to view the set of spatial relationships within the environment simultaneously, without actually navigating through the space; it provides a static view of the environment (see Uttal et al., 2006). The use of maps has been related to the development of allocentric spatial coding strategies (Uttal et al., 2006). Furthermore, the development of the ability to use allocentric coding has been associated with self-locomotion (Yan et al., 1998).

The second route learning task was presented using desktop virtual reality (VR) and thus represented a high level of physical realism. In contrast to the viewer-independent perspective presented in the spatial programming task, in this task participants viewed the environment from a viewer-centered/egocentric perspective. Participants were shown a route from A to B and asked to learn it. This perspective represents the prototypical manner in which we experience new environments; as we navigate, the relationship between ourselves and space is constantly changing, and landmarks are viewed sequentially. Desktop VR is ideally suited to this investigation because it neutralizes the demands of real-world locomotion, allowing a pure measure of spatial cognitive aspects of navigation.

The above two route learning tasks differ in their egocentric vs. allocentric representation of the environment, and the use of a map only in the spatial programming task. Landmark knowledge and route knowledge, as measured in both tasks, activate the parahippocampal gyrus (Wegman and Janzen, 2011) and the caudate nucleus respectively (Doeller et al., 2008). Allocentric coding and the development of configural knowledge, i.e., knowledge of the spatial relations between places within an environment activates the hippocampus, as part of the same interacting network (Doeller et al., 2008). Thus it is likely that the spatial programming task additionally activates the hippocampus. This is, of course, speculative without direct neural evidence.

We predicted poorer performance in the children with PD for both route learning tasks compared to a typically developing group. For the spatial programming task, this was based on the association between early locomotor experience and the development of allocentric coding (Clearfield, 2004). For the VR route learning task, this was based on previous reports of impaired spatial knowledge of large-scale environments in individuals with PD (Stanton et al., 2002; Wiedenbauer and Jansen-Osmann, 2006). Given the association between physical exploration and the development of both allocentric and egocentric spatial knowledge (Cornell et al., 2001; Oudgenoeg-Paz et al., 2015), as well as the impact of passive vs. active route learning on performance (Foreman et al., 1994), we predicted a further differentiation between children with PD who used a wheelchair vs. those who could walk independently, with the poorest performance predicted for the PD participants who used a wheelchair. This was based on the assumption that wheelchair users had relatively limited opportunity for independent exploration compared to non-wheelchair users. Due to the heterogeneity of neural damage in individuals with PD, we did not make predictions based on the neural activation of each spatial task.

We also included a memory element to the VR route learning task, in which participants were asked to recall landmarks along the route. Whilst this had a spatial element, it could be solved using visual recognition and so we did not predict a deficit in the PD participants on this measure.

## Materials and Methods

### Participants

For the mental rotation and spatial programming tasks, 51 typically developing children were recruited from mainstream schools in the United Kingdom (see Table 1). For the VR route learning task, in addition to the fifty-one TD children who completed the full battery of tasks, data was also included from TD children who had completed this task as part of a different study (Farran et al., 2019) bringing the total number of TD children to *N* = 122 for this task. The TD children ranged from 5 to 11 years, chosen to span the mental age range of the PD participants (which was lower than their chronological age, on account of their learning difficulties). This allows us to compare the performance of the PD group to what would be expected for their level of non-verbal ability, thus taking into account their learning difficulties.

**TABLE 1:** Participant details for the mental rotation and spatial programming tasks (mean and range).

**Group**	**Chronological age (years; months)**	**BAS3 matrices ability score^1^**	**BPVS raw score**	**Movement ABC checklist. Total Motor Score**
PD – wheelchair user (*N* = 32)	13;06 (5;11–18;02)	85.28 (58–139)	115.09 (50–164)	61.32 (19–87)
PD – no wheelchair use (*N* = 18)	13;10 (6;06–18;02)	98.22 (58–157)	112.28 (52–157)	19.00 (1–46)
TD (*N* = 51)^2^	8;10 (5;10–11;07)	118.18 (58–163)	123.20 (78-160)	NA

*^1^BAS3 Matrices ability scores are derived from the first item that was assessed and are equivalent to raw scores.*

*^2^One participant in the TD group did not complete the mental rotation task. In order for the range of BAS ability scores to be similar across the groups, the three participants with the lowest BAS matrices scores in the PD groups were excluded from the sample for mental rotation and Bee-Bot analyses.*

Fifty-three participants with PD (all with statements of special educational needs) were recruited from two special schools in the United Kingdom. All children with PD who were invited to take part met the criteria of being able to verbally communicate (some children supported this by signing or gesturing), having the ability to use the keys on a computer keyboard (some children used a large-keys keyboard), and all had normal or corrected to normal vision. One of the authors, who was also a teacher of the children with PD, also completed the Movement Assessment Battery for Children 2 - checklist (MABC2; Henderson et al., 2007) for each participant. The MABC2 checklist is a thirty-item checklist in which the respondent rates the child’s motor competence on a 4-point scale (0, 1, 2, or 3). The questions refer to motor skills such as self-care skills, classroom skills, recreational skills, and ball skills. A total motor score is provided which is the sum of the thirty scores, with a higher score indicative of poorer motor performance. The MABC2-checklist correlates significantly with performance on the MABC2 test (*r* = 0.38; *p* < 0.001; Schoemaker et al., 2012) and has high construct validity (Cronbach’s α: 0.94; Schoemaker et al., 2012). All participants completed the Matrices subtest of the British Ability Scale 3 (BAS3; Elliot and Smith, 2011) as a measure of non-verbal ability and the British Picture Vocabulary Scale III as a measure of verbal ability (Dunn and Dunn, 2009).

The children with PD were divided into two groups: (1) wheelchair users (used wheelchairs every day and for most of the day) and part-time wheelchair users (used wheelchairs for part of the day or the week); and (2) non-wheelchair users (although some of this group may have used wheelchairs at an earlier age) (see Table 1). A large proportion of the children with PD had received a diagnosis of Cerebral Palsy; *N* = 33/34 (97%) in the PD wheelchair group, and *N* = 6/18 (33%) in the PD no wheelchair group. Individuals with Cerebral Palsy have known deficits in visuo-spatial perception (e.g., Ego et al., 2015; Critten et al., 2018, 2019). The extent to which these deficits are independent of their motor impairment is not possible to ascertain. However, given that Cerebral Palsy is a lifelong disorder caused by cortical damage before, during or soon after birth, and the known developmental association between motor and spatial domains, it is highly likely that early disordered motor development in these participants has an impact on the development of spatial cognition (see Stanton et al., 2002), similar to that of an individual with a lifelong motor deficit without a diagnosis of cerebral palsy.

Ethical approval was obtained from the University Ethics Committee. Parental written consent and the children’s verbal consent were obtained prior to testing. Children were tested individually in quiet areas or rooms in 20–30 min sessions. For each task, participants were given no help during the tasks beyond the standardized instructions. As this was part of a larger battery of tasks, children took part in approximately six sessions. The additional TD children who received the VR navigation task, the BAS3 matrices and BPVS were presented with these tasks under the same conditions (the same 17 inch laptop was used for VR navigation task, task administration was identical, and testing took part in a quiet area of the school within a 30-min testing session, as part of a larger battery of tasks).

### Design and Procedure

#### Mental Rotation Task

**FIGURE 1:**
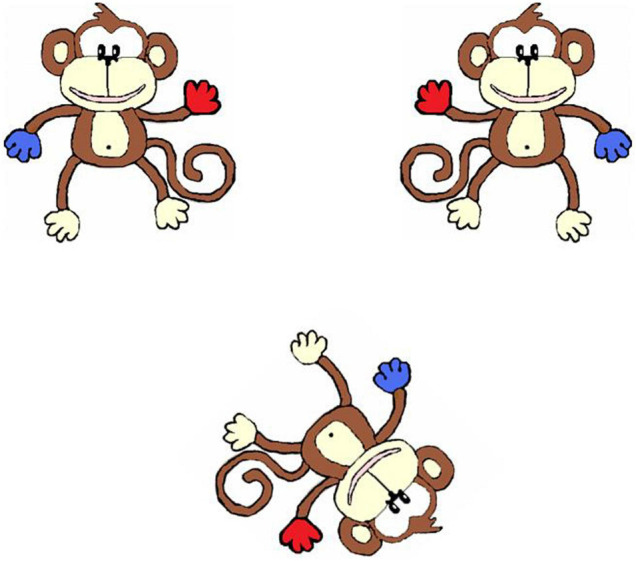
Example mental rotation stimuli.

This task, from Broadbent et al. (2014b), was presented on a 17 inch laptop computer. Participants viewed two mirror imaged monkeys on the top half of the screen and the test monkey on the bottom half of the screen (Figure 1) and were asked to choose which of the two monkeys on the top half of the screen matched the monkey on the bottom half of the screen. They responded by pressing one of two keys on the keyboard. A large-keys keyboard was available for children who found the laptop keys difficult to access, and two participants chose to answer by pointing, and their choices were inputted for them. There were 6 practice trials followed by 32 experimental trials. In the practice trials, the test monkey was rotated 0° (four trials), 45° (one trial), or 90° (one trial). The practice block was repeated if participant made any errors on these trials. No feedback was given for experimental trials, but motivation language was used at the end of the task such as “Well done.” In the experimental trials, the test monkey was rotated at 0°, 45°, 90°, 135°, or 180°. Accuracy was recorded.

#### Spatial Programming Task

**FIGURE 2:**
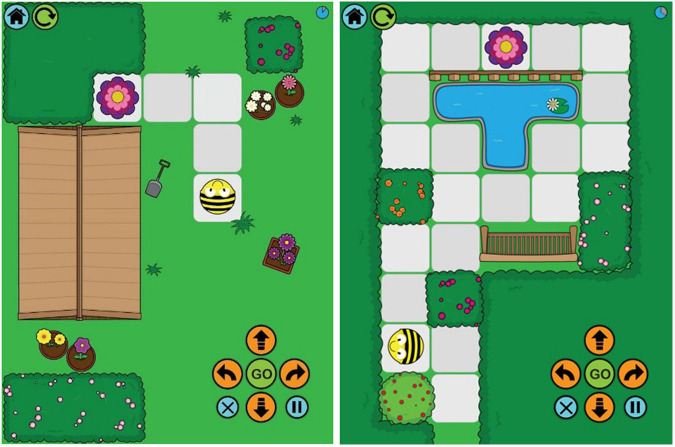
Bee-Bot app showing Routes 3 and 9. Images published with permission from TTS group (https://www.tts-group.co.uk/).

The Bee-Bot app^1^ was presented on an iPad. There are twelve route planning games on the app, starting with a very simple route for the Bee-Bot to reach a flower (Figure 2). The routes gain in complexity and some routes have more than one algorithm to complete them. The first two routes were used as practice routes, and Routes 3–9 were used as experimental routes (seven routes). Participants were told that they would need to program the Bee-Bot to move it from the start along the route to the flower using the arrow keys in the corner of the screen. Participants were asked to program all moves before they started the Bee-Bot on the route by pressing the GO key. The experimental trials commenced once participants had passed the two practice trials.

Participants were told that if they made an error, they would be allowed to have another go. If participants perceived that they had made an error, motivational language was used (e.g., “Good effort”) and they were encouraged to try again. There were a maximum of five trials for each route, and if the child did not complete a route correctly within the five trials, then the task finished. The task was scored as the number of routes attempted by the children (route accuracy: max = 7). We also recorded the number and type of errors made by participants. A correct programming algorithm included two types of commands; forward displacement of the Bee-Bot and left or right 90-degree rotation of the Bee-Bot. Errors scores were coded as a proportion of errors for that command type within the route, e.g., if there were two rotation commands in a route, an error of one would give a proportion of 0.5. The mean proportion error score across the number of routes attempted, for each error type, was used as the dependent variable.

#### VR Navigation Task

The VR navigation task was from Farran et al. (2012). Virtual environments (VEs) were created using Vizard^2^ and presented on a 17 inch laptop computer. The VEs displayed brick-wall mazes which could be navigated using the arrow keys on the keyboard. Preceding the experimental maze, the participants watched the experimenter navigate a simple corridor that included two turns. Then they practiced navigating along the corridor. If participants had difficulty controlling their navigation, they were given another attempt.

**FIGURE 3:**
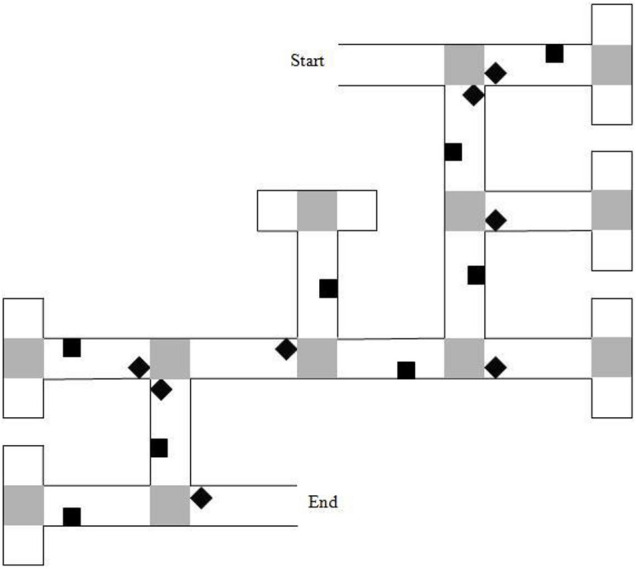
Map of the 6-turn maze layout. Gray squares represent “pebble” texture that was featured at junctions and at the end of cul-de-sacs. Black diamonds indicate junction landmarks. Black squares indicate path landmarks. Reproduced from: Farran et al. (2012).

The experimental VE displayed a brick-wall maze with 6 junctions, each leading to two paths, one correct and one incorrect. The 6 correct choices constituted two left, two right, and two straight-ahead choices. A map of the maze layout is shown in Figure 3. Each incorrect path choice ended in a cul-de-sac and looked like a T-junction when viewed from the preceding junction. Sixteen unique landmarks featured throughout the maze and featured equally on the left and right of the paths. Eight of the landmarks were near to junctions (‘junction landmarks’). Eight of the landmarks were not near to junctions (‘path landmarks’). Landmarks were selected from a range of categories (e.g., animals, tools, furniture) for their high verbal frequency (Morrison et al., 1997) and for being easy to recognize. A gray duck was shown at the end of the maze. On approaching the duck, the game ended.

##### Route learning task

Participants were instructed to learn a single six junction route through a maze. The experimenter showed the participant the correct route through the maze by using the arrow keys on the keyboard to navigate and told the participant to watch, because it would be their turn to navigate next. After the experimenter demonstration, the participant attempted to walk the correct route from start to finish using the arrow keys. A large-keys keyboard was available for those children who found the laptop keys difficult to access. If the participant selected an incorrect path, they reached a cul-de-sac and could self-correct by turning around. If a participant was going backwards to the start of the maze, they were directed back to the junction where they made the error. On reaching the gray duck (i.e., on completing the route) the trial terminated. Motivational language was used throughout to maintain participant concentration.

Each walk through the maze from start to finish of the route was labeled a learning trial. The criterion for having learnt the route was the successful completion of two consecutive learning trials from start to finish without error. If participants did not meet this criterion after ten learning trials, the task was stopped. The cumulative number of errors across learning trials was recorded; this was used as the dependent variable. An error was defined as a deliberate incursion down an incorrect path; if the participant corrected his/her course before reaching half-way down an incorrect path section, no error was counted.

##### Landmark recall task

After the participant had learnt the six junction route to criteria, they completed a landmark recall task. Participants were shown the same maze but with all landmark objects shown as red balls. The experimenter navigated, stopping at each junction to point out the red ball(s). Participants were asked to recall what object the ball had been when they were navigating the route. On providing an answer, the participant was shown a visual image of the correct answer on another computer screen as feedback, i.e., the landmark in its correct location. This feedback was given to eliminate any dependency between their answers (e.g., if the participants answered incorrectly at one location, without feedback they might not have used that landmark label again, or their incorrect answer might have negatively influenced their subsequent performance if they had recalled the landmarks in sequence). This was conducted for all 12 landmarks that were visible from the correct path. Eight of these landmarks were on the correct path, there were also four landmarks that could be viewed straight ahead before a correct turn to the left or right was executed).

To ensure that the verbal labels used by the participants in the landmark recall task could be coded accurately (e.g., a participant might use the word “light” for “streetlamp”), after the landmark recall task, participants were shown images of each of the 16 landmarks and were asked to name them. This information was then used to retrospectively facilitate the scoring of the landmark recall task.

## Results

### Overview of Analyses

Where suitable, the data is analyzed using developmental trajectory analysis (Thomas et al., 2009). Developmental trajectory analysis does not require the individual matching of the participants and goes beyond determining differences in group means, to ascertain whether the trajectory of performance across the range of mental ages of each group differs at the onset of the trajectory (the youngest mental ages measured) or the rate of development. For developmental trajectory analysis to be meaningful, it is important that a measure of mental age (in this study, BAS3 matrices ability score) correlates with the task dependent variables. This was the case for the mental rotation and spatial programming tasks, but not the VR navigation task. For the VR navigation task, comparison was by group means instead.

Developmental trajectory analyses were ANCOVAs with Group as the between-participant factor and BAS3 matrices ability scores as the covariate. We chose BAS3 matrices ability score (equivalent to raw score) as our measure of mental age because it is a measure non-verbal ability and thus represents ability within the same domain as the tasks of interest. BAS3 matrices ability score was rescaled so that the *X*-axis crossed the *Y*-axis at the lowest BAS matrices score (a score of 58) of the participants. That is, we subtracted 58 from all BAS matrices scores for these analyses. This does not change the analyses but is easier to interpret because the starting point for the trajectories is at zero. The ANCOVA model included interaction terms between the BAS3 matrices covariate and Group. This was used to indicate whether spatial ability developed at a different rate for each group, with respect to non-verbal ability.

The mental rotation variables were broadly normal (Kolmogorov–Smirnov, *p* > 0.05). Spatial programming and VR navigation variables were largely not normally distributed (Kolmogorov–Smirnov, *p* < 0.05). Because ANOVA is robust to violations of assumptions of normality, parametric analyses were applied (Blanca et al., 2017) with one exception, maze error. For this variable, responses were skewed toward zero, and thus non-parametric analyses were conducted. For associational analyses, parametric and non-parametric analyses were applied for normal and non-normal distributions respectively.

### Mental Rotation

**FIGURE 4:**
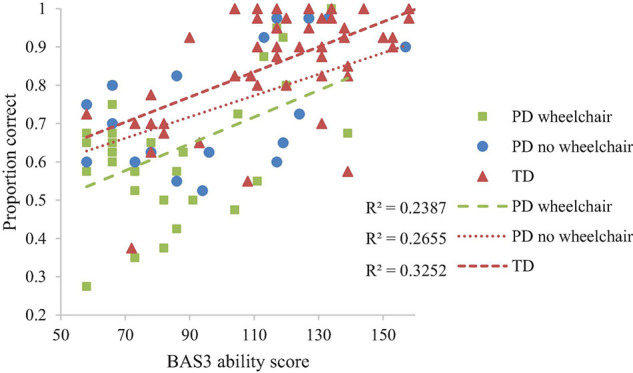
Developmental trajectory of mental rotation performance.

Developmental trajectory analysis was conducted on the proportion of correct answers with degrees of rotation (0°, 45°, 90°, 135°, 180°) as a within-participant factor and Group as a between-participant factor. This revealed the anticipated main effect of rotation (decrease in accuracy with increasing degrees of rotation), reported as a linear contrast, *F*(1,94) = 18.94, *p* < 0.001, η_*p*_^2^ = 0.17. The effect was consistent across participant groups, *F* < 1. There was no group difference in proportion correct at the lowest level of non-verbal ability (i.e., at the intercept of the trajectories), *F*(2,94) = 2.529, *p* = 0.085, η_*p*_^2^ = 0.051). However, because this effect was marginal we had reason to explore it. This revealed a lower proportion correct at the lowest level of non-verbal ability in the PD wheelchair group compared to the TD group only (*p* = 0.036; other comparisons; *p* > 0.05; see Figure 4). There was no interaction between non-verbal mental age and group, which is indicative of similar rates of development across groups, *F* < 1. BAS3 matrices score (non-verbal mental age) was significantly related to proportion correct, *F*(1,94) = 32.079, *p* < 0.001, η_*p*_^2^ = 0.254. All other interactions with BAS3 matrices score were non-significant, *p* > 0.05 for all.

### Spatial Programming

#### Spatial Programming Route Accuracy

**FIGURE 5:**
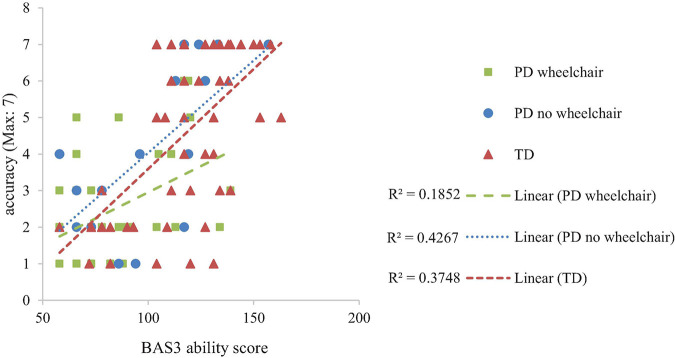
Developmental trajectory of spatial programming accuracy.

Developmental trajectory analysis on the number of routes attempted (route accuracy, maximum = 7) with Group as a between-participant factor demonstrated no group difference at the lowest level of non-verbal ability, *F* < 1 and similar rates of development, *F*(2,95) = 1.379, *p* = 0.357, η_*p*_^2^ = 0.296 across the groups. BAS3 matrices score was significantly related to Bee-Bot route performance, *F*(1,95) = 39.875, *p* < 0.001, η*_*p*_^2^* = 0.296 (Figure 5).

#### Spatial Programming Errors

**FIGURE 6:**
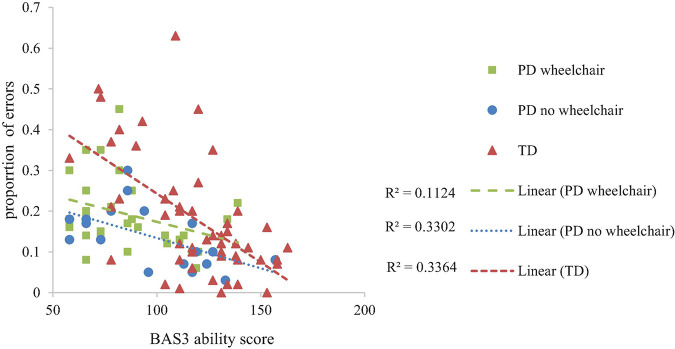
Developmental trajectory of proportion of spatial programming errors per route attempted.

Developmental trajectory analysis on proportion error scores, with a within-participant factor of Error Type (forward errors, turn errors) and Group as a between-participant factor demonstrated a group difference at the lowest level of non-verbal ability, *F*(1,95) = 7.525, *p* = 0.001, η_*p*_^2^ = 0.14 and an interaction between non-verbal ability and group, which is indicative of different rates of development, *F*(2,95) = 3.20, *p* = 0.045, η*_*p*_^2^* = 0.063 across the groups. This was accounted for by significantly more errors at the intercept in the TD group compared to both of the PD groups (TD vs. PD wheelchair, *p* = 0.002; TD vs. PD no wheelchair, *p* = 0.002; PD no wheelchair vs. PD wheelchair, *p* = 0.417), and a steeper improvement with development in the TD group compared to the PD wheelchair group (TD vs. PD wheelchair, *p* = 0.041; TD vs. PD no wheelchair, *p* = 0.076; PD no wheelchair vs. PD wheelchair, *p* = 0.853). The slopes of the trajectories for each error type did not differ, *F*(1,95) = 1.09, *p* = 0.30, η*_*p*_^2^* = 0.011, and this pattern was consistent across groups, *F*(2,95) = 2.00, *p* = 0.14, η*_*p*_^2^* = 0.040. BAS3 matrices demonstrated a significant association with spatial programming errors, *F*(1,95) = 22.39, *p* < 0.001, η*_*p*_^2^* = 0.19. Figure 6 illustrates developmental trajectories collapsed across error type.

### Navigation

A larger TD group was employed for this task, which enabled comparison with TD groups in different age ranges (Table 2). The PD groups had a similar level of BAS3 matrices ability score to the TD 5–7 year-olds (PD wheelchair vs. TD 5–7: *p* = 0.410; PD no wheelchair vs. TD 5–7: *p* = 0.945) and a lower level of BAS3 matrices ability score than the TD 8–9 year-olds and the TD 10–11-year-olds (*p* < 0.05 for all).

**TABLE 2:** Participant details for the VR navigation task (mean and range).

**Group**	**Chronological age (years; months)**	**BAS3 matrices ability score^1^**	**BPVS raw score**	**Movement ABC checklist Total Motor Score**
PD – wheelchair user (*N* = 34)	13;06 (5;11–18;02)	82.91 (41–139)	115.06 (50–164)	61.03 (19–87)
PD – no wheelchair use (*N* = 19)	13;10 (6;06–18;02)	95.58 (48–157)	112.26 (52–157)	19.89 (1–46)
TD 5-7 years (*N* = 44)	6;07 (5;10–7;11)	91.27 (37–131)	99.36 (69–136)	NA
TD 8-9 years (*N* = 47)	8;10 (8;01–9;10)	123.64 (95–154)	125.87 (92–154)	NA
TD 10-11 years (*N* = 31)	10;09 (10;03–11;07)	140.55 (104–177)	141.71 (97–160)	NA

*^1^BAS3 Matrices ability scores are derived from the first item that was assessed and are equivalent to raw scores.*

#### Maze Errors

Kruskal–Wallis ANOVA with a dependent variable of maze error was conducted with Group as the between participant factor. This demonstrated a main effect Group, χ^2^(4) = 11.753, *p* = 0.019. Mann–Whitney paired comparisons demonstrated that this was due to: (1) the PD groups making more errors than the TD 10–11 year-olds (PD wheel chair vs. TD 10–11 years, *p* = 0.008; PD no wheelchair vs. TD 10–11 years, *p* = 0.015); and (2) developmental progression across the TD groups (TD 5–7 years vs. TD 10–11 years, *p* = 0.003; TD 8–9 years vs. TD 10–11 years, *p* = 0.044) (all other comparisons, *p* > 0.05) (Table 3).

**TABLE 3:** Cumulative number of errors made on the VR navigation task across learning trials.

**Group**	**Median (range)**
PD wheelchair	2 (0–14)
PD no wheelchair	2 (0–12)
TD 5–7 years	2 (0–7)
TD 8–9 years	1 (0–9)
TD 10–11 years	1 (0–7)
	

#### Landmark Recall

**FIGURE 7:**
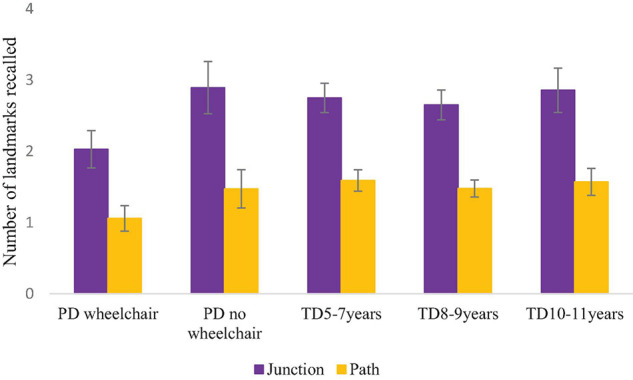
Mean (S.E.) number of landmarks recalled per group.

ANOVA of the number of junction and path landmarks that were correctly recalled was carried out, with a between-participant factor of Group and a within-participant factor of Landmark Type (path, junction). This demonstrated no difference in the number of landmarks recalled across groups, *F*(4,166) = 2.093, *p* = 0.084, η*_*p*_^2^* = 0.048 (Tukey pairwise comparisons were non-significant for this marginal effect: *p* > 0.05 for all). There was a main effect of landmark type due to stronger recall of junction than path landmarks, *F*(1,166) = 159.463, *p* < 0.001, η*_*p*_^2^* = 0.490, which did not interact with group, *F* < 1 (Figure 7).

### Associations Between Motor Ability and Spatial Competence

We were also interested in how performance on the M-ABC checklist correlated with each of our spatial dependent variables. M-ABC checklist data is available for the two PD groups only, and so the correlation matrix below does not include the TD group. As shown in Table 4, there were no significant correlations between motor score and spatial competence.

**TABLE 4:** Correlations between Movement ABC checklist Total Motor Score and spatial variables, for each PD group.

	**Mental rotation**	**Spatial programming**		**VR navigation**	
		**Accuracy**	**Errors**	**Maze errors**	**Landmark recall**
PD – wheelchair user (*N* = 34)	*r* = –0.30, *p* = 0.09	^+^*r* = –0.23, *p* = 0.20	^+^*r* = 0.28, *p* = 0.20	^+^*r* = –0.30, *p* = 0.09	^+^*r* = –0.07, *p* = 0.69
PD – no wheelchair use (*N* = 19)	*r* = 0.08, *p* = 0.75	*r* = 0.03, *p* = 0.91	*r* = 0.004, *p* = 0.99	^+^*r* = 0.05, *p* = 0.85	*r* = –0.322, *p* = 0.18

*^+^Indicates Spearman correlations. All remaining are Pearson correlations.*

## Discussion

The current study had two aims. The first aim was to investigate the relationship between motor ability and spatial competence by working with participants for whom motor ability is impaired. The second aim was to investigate whether this relationship differed for those who were wheelchair users and potentially limited in opportunities for independent exploration, compared to those who could walk independently. All participants with PD had a statement of special educational needs (e.g., moderate learning difficulties, epilepsy). This was evident in their level of non-verbal ability, which was commensurate with that of TD 5- to 7-year-olds.

We predicted that the PD groups would show impaired spatial ability on all three tasks. We also predicted a differentiation in performance between the two PD groups for all three spatial tasks, with the PD wheelchair group finding the tasks harder than the PD no wheelchair group, on account of differences in their opportunities for independent exploration. We found that level of impairment in the PD groups across tasks was broadly akin to their level of non-verbal ability (note that the PD groups had poor non-verbal ability). This demonstrates that spatial ability is poor (i.e., it is not age-appropriate), but that in the context of the learning difficulties of these individuals, it does not represent a specific area of weakness. The one exception to this was performance of the PD wheelchair group on the mental rotation task, where performance was lower than expected for their level of non-verbal ability. Mental rotation taps into intrinsic spatial skills, whilst the two spatial route tasks tap into extrinsic spatial skills (Uttal et al., 2013; Newcombe, 2018). Precisely why performance on the mental rotation task and/or intrinsic spatial skills would show a specific impairment relative to the two spatial route tasks and/or extrinsic spatial skills is difficult to determine. The difference could relate to the neural activation of motor areas of the brain in the mental rotation task specifically (Zacks, 2008). However, this is a tentative explanation given the known heterogeneity in neural deficit in individuals with physical disability and learning difficulties (e.g., Stadskleiv et al., 2017).

The overall pattern of performance observed could also reflect differences in the sensitivity and specificity of the route learning tasks. The VR navigation task relied on landmark knowledge and route knowledge and thus did not draw on the more sophisticated configural knowledge. Navigational tasks that rely on configural knowledge, an ability which develops in typically developing children between the ages of 5 and 10 years (Bullens et al., 2010; Broadbent et al., 2014a), might have been more sensitive to group differences. Furthermore, neither of the route learning tasks are pure measures of spatial ability. Route knowledge tasks also draw on executive function skills (Purser et al., 2012) and we discuss below that, for the spatial programming task, the working memory and attention demands of the task might explain the pattern of errors of the two PD groups. Limitations in working memory and attention, on account of learning difficulties could thus overshadow any differences between the two PD groups in spatial competence. The pattern of spatial performance in the PD wheelchair group is discussed further within the context of each task below.

We also predicted that performance on the landmark recall task would not be an area of deficit for the PD groups. This was the case. In fact, there were no group differences on this task, demonstrating that the mechanisms tapped into on this task (object memory) were not impacted by either physical disability or the participants’ learning difficulties. Although, note that the lack of evidence in progression in the three TD groups could also suggest that this measure was not sensitive to developmental differences. Performance on each task is discussed in turn below.

Performance on the mental rotation task demonstrated a linear decrease in accuracy with increasing degrees of rotation for all groups. This pattern was expected for the TD group (e.g., Farran et al., 2001). The presence of this typical pattern for both of the PD groups suggests that the PD groups were capable of performing mental rotation and approached the task in a typical manner. Despite this, the PD groups performed at a lower level than expected for their chronological age (mean: 13 years), and at a level commensurate with their level of non-verbal mental age. A lower level of performance was observed in the PD wheelchair group, compared to the TD group from the lowest level of non-verbal ability and remained consistently low throughout the range of non-verbal abilities, as indicated by the similar rate of development to the TD group. In other words, across the range of non-verbal abilities that we examined, the PD wheelchair group was consistently and to the same degree poorer than the TD group on the mental rotation task, suggesting delayed but parallel development. In contrast, for the PD no wheelchair group, performance was on a par with the developmental trajectory of the TD group and therefore as expected for their level of non-verbal ability. Thus, any deficit in mental rotation ability in this group appears to be attributable to having learning difficulties (indexed here by non-verbal ability), rather than motor impairments. Note, these group comparisons were explored based on a marginal interaction effect and so should be considered cautiously.

The PD wheelchair group are likely to have limited experience of exploration and limited experience of actively moving through their environment. This could have a developmental cascading impact on the development of their ability to perform mental rotation. This is supported by Oudgenoeg-Paz et al. (2015) who demonstrated that exploration in TD toddlers was longitudinally predictive of their performance on a block construction task (a task which involves mental rotation; Farran et al., 2001). It is also noteworthy from the MABC-checklist scores that the PD wheelchair group had more severe motor impairment than the PD no wheelchair group. This was the case across all subsections of the checklist (Table 5), including sections A1 and A2 which included fine motor items. It is possible that this broad difference in motor competence between the PD groups, rather than or in addition to their experience of independent exploration, can explain why mental rotation was impaired in the PD wheelchair group relative to their non-verbal ability. Whilst this is not statistically supported by the correlational analyses which indicated no significant associations between motor ability and spatial competence, the relationship does show a medium effect size for this group (Cohen, 1988) and the lack of significance could reflect a lack of power for these analyses. In support of a broad motor-spatial relationship, Soska et al. (2010) report a relationship between the fine motor skills required for visual-manual exploration and small-scale spatial abilities in 4.5–7.5 months-old infants. Further support is offered from evidence that mental rotation draws on mechanisms that are common to motor activity at neural and behavioral levels (Parsons et al., 1995; Zacks, 2008), supporting a direct impact of motor impairment on performance on this task for the PD wheelchair group. Further research with a larger participant group is required to determine the motor-spatial association in this context.

**TABLE 5:** Movement ABC checklist profile of scores for the PD groups (a higher score indicates higher severity).

	**PD wheelchair user (*N* = 34)**	**PD no wheelchair use (*N* = 19)**	**Group comparison (*t*-test)**
A1 (max: 15)	8.44 (0–15)	2.16 (0–7)	*p* < 0.001
A2 (max: 15)	9.24 (0–15)	2.32 (0–7)	*p* < 0.001
A3 (max: 15)	11.35 (5–14)	2.74 (0–8)	*p* < 0.001
B1 (max: 15)	9.41 (3–13)	1.79 (0–7)	*p* < 0.001
B2 (max: 15)	11.55 (6–15)	7.21 (1–11)	*p* < 0.001
B3(max: 15)	11.32 (5–15)	3.68 (0–7)	*p* < 0.001
Total	61.32 (19–87)	19.89 (1–46)	*p* < 0.001

*A1, Static/Predictable Movement, Self-Care Skills; A2, Static/Predictable Movement, Classroom Skills; A3, Static/Predictable Movement, PE/Recreational Skills; B1, Dynamic/Unpredictable Movement, Self-Care/Classroom Skills; B2, Dynamic/Unpredictable Movement, Ball Skills; B3, Dynamic/Unpredictable Movement, PE/Recreational Skills.*

For both of the PD groups, performance on the navigation task was lower than the level of 10- to 11-year-old TD children, despite the age range of the PD groups spanning from 5 to 18 years. This level of navigation ability is broadly in line with the level of non-verbal ability of the two PD groups, which was similar to that of the TD 5- to 7-year-old group. The association between motor ability and performance on the VR navigation task showed a medium (albeit non-significant) effect size for the PD wheelchair group. Whilst this could be taken to suggest some impact of their motor impairment on navigation performance, the lack of group difference in navigation performance between the two PD groups suggests that the physical disabilities of the PD groups were not the limiting factor, but rather it was their learning difficulties. At first blush, this appears to contrast to previous reports of impaired navigation in people with physical disabilities (Stanton et al., 2002; Wiedenbauer and Jansen-Osmann, 2006). However, on a closer look, it simply reflects differences in the matching procedures across the studies. Stanton et al. (2002) did not measure IQ (all participants had cognitive performance in the ‘normal’ range) and matched participants by Chronological Age. Thus, their PD group performed at a lower level on a navigation task than expected for their chronological age, which is largely consistent with the current study. Furthermore, Stanton et al. (2002) also used a developmentally more sophisticated measure of navigation, which might had differentiated the groups more than the current measure of navigation. Given that a large proportion of their sample had a diagnosis which implicates poor visuospatial cognition (Cerebral Palsy or Spina Bifida), without cognitive data it is difficult to disentangle the extent to which this contributed to their navigation performance. Wiedenbauer and Jansen-Osmann (2006) report data from children with Spina Bifida and TD controls. Their groups were matched on Chronological Age and Verbal IQ and thus the Spina Bifida group had lower non-verbal IQ than the TD control group. As such, the deficit in navigation that they report is relative to their Chronological Age and not their (lower) non-verbal ability; our data are also broadly consistent with this pattern of findings, as we observed a deficit relative to Chronological Age. One might argue that by comparing spatial performance in our sample to their level of non-verbal mental age, we are risking matching away any group differences. Whilst this is a risk, it is the most appropriate way to account for the cognitive learning difficulties of our PD samples. Furthermore, the use of developmental trajectories and error analyses in this study has enabled us to capture additional information in relation to development, individual differences and task approach.

The pattern of performance on the navigation task demonstrated that all groups had stronger recall of landmarks at junctions than landmarks on other parts of the path sections. This is in line with our predictions and suggests that all children were using a landmark strategy when learning the route, i.e., they understood that landmarks at junctions were relatively more useful for route learning than other landmarks. This strategy is consistent with the literature on the typical development of route learning (e.g., Farran et al., 2012), and appears to be robust to atypical development as it has been observed in several atypical groups including Williams syndrome (Farran et al., 2012) and children with Attention Deficit Hyperactivity Disorder (ADHD) (Farran et al., 2019). Consequently, despite having both physical disabilities and learning difficulties, these participants appeared to be able to encode landmarks effectively, and use them as a tool when navigating.

The pattern of performance of the PD groups on the spatial programming task differed from that of the TD group. For both PD groups, the number of routes attempted was in line with that expected for their level of non-verbal mental age and showed a typical rate of development. This was, however, coupled with group differences in the error patterns which suggests that the PD groups were approaching the task in a different manner to the TD group. Developmentally, at the lowest level of non-verbal ability, the TD group had higher proportion error scores than both PD groups, even though they were more successful in progressing through the routes. There are a number of reasons for this finding. A high proportion of errors could indicate a difficulty in perspective taking. For example, if the Bee-Bot is facing right, and it needs to move upwards on the iPad, the participant must determine that this requires a 90° left turn, i.e., they need to view the turn from the perspective of the Bee-Bot and not themselves. Given that perspective taking is a relatively late spatial skill to develop (Frick et al., 2014), this might have impacted the TD group more than the PD group who had more years of experience and perhaps more exposure to allocentric representations of space. The relatively late development of perspective taking (Frick et al., 2014) and processing allocentric representations (Bullens et al., 2010; Broadbent et al., 2014a) could explain why the TD group exhibited a high number of errors at the lowest level of non-verbal ability. This contrasts to the other spatial skills measured in this study, such as mental rotation and route knowledge, which are available from at least five years in typical development (e.g., Lingwood et al., 2015). Furthermore, due to the threshold procedure employed, the TD group were exposed to a broader range of routes, and so encountered relatively more of the difficult routes (which necessarily included more changes in perspective) than the PD groups. If perceptive taking and/or allocentric coding was more problematic for those with lower non-verbal ability, this would be compounded by exposure to a larger range of routes, as observed in the TD group. The group difference at the trajectory intercept was coupled with a steeper rate of development for the TD group relative to the PD groups, which meant that the TD group caught up with the PD groups as non-verbal ability increased. This difference in the rate of development between the TD and PD groups might reflect differences in the performance limitations of each group. If the TD group are initially failing due to poor perspective taking and/or poor allocentric knowledge, their rate of development might be related to the development of these spatial skills. The PD group might have an initial advantage in these spatial skills due to their higher chronological age and level of experience with map-like representations. However, other factors might limit their progression such as juggling the spatial demands with more domain general demands such as working memory and attention, skills which might be limited in these groups due to their general learning difficulties. This might have led participants to make mistakes such as miscounting the number of paving slabs, losing where they are on the route when planning their algorithm, or forgetting the function of the buttons (e.g., understanding that the turn function programs the Bee-Bot to turn within their own square rather than moving forward one square when it turns). These kinds of limitations could be more confounding across the range of non-verbal abilities, hence the shallower rate of development in these groups. These kinds of limitations might also explain why there was no difference in performance between the two PD groups. These tentative suggestions require further research which take into account the involvement of working memory and attention processes in this task.

Whilst our findings are consistent with the conclusion that physical disability *per se* does not necessarily have a broad impact on spatial competence, it is difficult to disentangle the bi-directional developmental influence of physical disabilities and learning difficulties when both are present from birth, as in our sample. A large proportion of our sample had a diagnosis of Cerebral Palsy, which is known to present with deficits in visuospatial perception alongside motor difficulties (although note evidence for heterogeneity in visuospatial perception in Cerebral Palsy; Critten et al., 2019). We cannot rule out that any atypicalities observed in the current sample are driven by limitations in visuospatial perception that are associated with Cerebral Palsy. However, all of our PD participants had a lifelong disorder and given the known interacting developmental trajectories of the spatial and motor domains (e.g., Yan et al., 1998; Clearfield, 2004; Jansen and Heil, 2010; Oudgenoeg-Paz et al., 2015; Farran et al., 2019), further research is required to determine any differentiated impact of a diagnosis of Cerebral Palsy, in individuals with PD and a learning disability, on spatial competence. We predict that a lifelong physical disability in *any* individual could impact the spatial domain.

To summarize, we have shown across three different spatial tasks that children with PD and learning disabilities perform lower than an age-appropriate level, but for the most part, at the level expected for their level of non-verbal mental age. Mental rotation was one exception to this finding; a skill that was particularly problematic for the children who relied on a wheelchair. We also observed unusual error patterns in both PD groups on the spatial programming task. Whilst it appears that having a physical disability did not always impact the development of spatial cognition over and above any general learning difficulties in our groups, there were indications of some minor, but potentially significant impacts of having a physical disability on spatial cognition. This highlights the importance of enabling active exploration for individuals with PD, particularly for those who are wheelchair users; evidence supports the importance of learning spatial layouts using free-choice and active exploration, over and above whether children locomote or use a wheelchair (Foreman et al., 1994).

## Data Availability Statement

The dataset presented in this study can be found in the following online repository: https://osf.io/75skq/.

## Ethics Statement

This study was reviewed and approved by UCL Institute of Education. Written informed consent to participate in this study was provided by the participants’ legal guardian/next of kin.

## Author Contributions

EF, VC, and DM conceived of the initial study design. VC collected the data from the Physical Disability participants. EC collected the data from Typically Developing participants. EF analyzed the data and wrote the manuscript. All the authors read, contributed to and approved the final manuscript.

## Conflict of Interest

The authors declare that the research was conducted in the absence of any commercial or financial relationships that could be construed as a potential conflict of interest.

## Publisher’s Note

All claims expressed in this article are solely those of the authors and do not necessarily represent those of their affiliated organizations, or those of the publisher, the editors and the reviewers. Any product that may be evaluated in this article, or claim that may be made by its manufacturer, is not guaranteed or endorsed by the publisher.

## Footnotes

1
https://www.tts-group.co.uk/

2
http://www.worldviz.com
